# The Sex Data Gap Within Implantable Cardioverter Defibrillator (ICD) Studies: A Retrospective Study of Literature From 1980 Until 2022

**DOI:** 10.7759/cureus.42139

**Published:** 2023-07-19

**Authors:** Peter M Magnusson, Kaitlyn R Morell, Jonelys Lazo, Ariella Listig, Joseph V Pergolizzi

**Affiliations:** 1 Center for Research and Development (CFUG), Uppsala University, Gävle, SWE; 2 Clinical Research, NEMA Research, Naples, USA

**Keywords:** sex data gap, chronic heart failure, sudden cardiac death (scd), cardiac therapy, implantable cardioverter-defibrillator (icd)

## Abstract

Implantable cardioverter defibrillators (ICDs) are a form of cardiac therapy used to prevent death in patients at risk for sudden heart failure. Using 100 articles from the introduction of ICDs until now, a retrospective literature review was conducted. These studies were analyzed for sex disparity over the past 40 years. The difference in the number of male participants to female participants was statistically significant for both the earlier and later study groups (p = 0.0014 and p = 0.0004 respectively), indicating a significant and consistently lower number of females in ICD research over time. This review shows no significant difference in the sex disparity since the implementation of ICDs. Unfortunately, due to a gap in the literature, the reason for this disparity between the sexes in ICD literature can only be speculated. This disparity may be partly due to a lack of incentive and encouragement for women to participate in research.

## Introduction

Implantable cardioverter defibrillators (ICDs) are a form of cardiac therapy that have been developed since 1980 with the intent to prevent death in patients at risk for sudden cardiac death (SCD) [1]. ICDs are used to treat tachycardia or ventricular fibrillation by detecting irregular heart rates and delivering a shock to restore the heart’s natural rhythm [2,3]. Originally the ICD was used only on patients with a high risk of sudden death from arrhythmias and was not immediately accepted in the medical community [1,4]. Now ICDs are used in both primary and secondary prevention treatments for patients at risk for sudden cardiac death and with roughly 3 million people at risk for SCD, ICD research and evolution is an important field [5,6]. ICDs have evolved constantly since their introduction 50 years ago including a decrease in size, addition of additional therapy formats and advanced algorithms. Biventricular forms of ICDs were also invented allowing for the treatment of cardiac heart failure and ventricular tachyarrhythmias [7,8]. Despite the extensive advancements and research on ICDs since the 1980s, it is believed there is a disparity between the sexes in available data. This research was designed to analyze changes in the sex data gap from 1988 to 2022. 

## Materials and methods

Using the PubMed search engine, 100 studies on implantable cardioverter defibrillators were collected based on the following filters: free full text, clinical trial, and randomized controlled trial. The studies were split equally into two time ranges from 1988 to the present. The first time period ranged from February 12th, 1988 to February 12th, 2005 and the second ranged from February 13th, 2005 to February 12th, 2022. Next, the studies were placed into a randomizer website (https://www.randomizer.org) to narrow down all studies available to 50 per time period. The randomization tool was used not only to narrow down available studies but also to eliminate possible biases and allow for generalization of the results.

Studies were ruled out based on systematic factors including, not being focused solely on ICDs, being an incomplete study, containing multiple studies, being inaccessible, and finally if the article was a substudy. Seven studies were ruled out for the first time period (1988-2005) and 15 studies were ruled out for the second time period (2005-2022) leaving 43 studies and 35 studies, respectively. 

The remaining 78 studies were then analyzed for sex differences in participants throughout the course of ICD history. The total number of participants male and female were logged for each study.

## Results

Among the 78 studies there were a total of 41,060 participants with 8,696 (21.18%) female and 32,364 (78.82%) male. The 43 studies from 1988 to 2005 included a total of 15,908 participants with 3,200 (20.12%) females and 12,708 (79.88%) males. Finally, the 35 studies included in the time period 2005 to 2022 consisted of a total of 25,152 participants with 5,496 (21.85%) females and 19,596 (77.91%) males. 

The difference in the number of male participants to female participants was statistically significant for both the earlier and later study groups (p = 0.0014 and p = 0.0004 respectively), indicating a significant and consistently lower number of females in ICD research over time. A majority of studies for both periods had 10% to 20% females, followed by 20% to 30% females. Five studies in the first period (1988 to 2005) had 0% to 10% female participants whereas all studies in the second period (2005 to 2022) had more than 10% female participants. Only two studies in the later period contained 40% to 50% female participants whereas the earlier period had no studies with more than 40% female participants. There was one study from each time period where participants' sex could not be determined. Sex differences are broken down in Table 1.

**Table 1:** Breakdown of Sex Differences in ICD Research From 1988-2022 ICD: implantable cardioverter defibrillator

Studies % Female	Studies 1988-2022	% Of Studies
0.0-10.0%	0/35	0.00%
10.1-20.0%	15/35	42.86%
20.1-30.0%	13/35	37.14%
30.1-40.1%	Apr-35	11.43%
40.1-50.0%	Feb-35	5.70%
Undetermined	Jan-35	2.86%
	Studies 2005-2022	
0.0-10.0%	May-43	11.63%
10.1-20.0%	21/43	48.84%
20.1-30.0%	14/43	32.56%
30.1-40.0%	Feb-43	4.65%
40.1-50.0%	0/43	0.00%
Undetermined	Jan-43	2.33%

For the 1988-2005 period, the average number of male participants was 302.57 (81.59%) whereas the average number of female participants was 76.19 (18.41%). For the 2005-2022 period, the average number of male participants was 578.12 (76.88%) whereas the average number of female participants was 161.65 (23.14%). Despite changes in legislation through these decades, there is very little change in the disparity between male and female ICD research participants. 

## Discussion

With one in every five female deaths being due to underlying heart disease, research on intervention and treatment options are necessary [9]. However, based on the data above females are represented disproportionately compared to their male counterparts. Only one study out of 78 included reasoning for excluding female participants, which excluded women of childbearing age not using medically prescribed contraceptives (Study 67: 1988-2005). There are two main factors that are speculated to be behind this disparity but without direct indications the true reasoning remains unknown. 

First, women may be less likely to enroll in these studies for a multitude of reasons. Over time societal norms have shifted but women are still expected to maintain a home life and work life leaving them with little time to be involved in a study. Women also often report a struggle to have their health and symptoms taken seriously by physicians [10]. This may discourage women from not only participating in studies but from seeking intervention in the first place. Studies have shown that males and females who have undergone similar surgeries with other controlled factors, males were more likely to receive pain medication post-operation [11,12]. Another study found women were less likely to be admitted to the hospital for chest pain than men [13]. The Yentl Syndrome, based on a case study, hypothesizes when women present like men with CHD they are more likely to be treated like men and because women do not typically present the same symptoms they are more likely to be undertreated [14]. Another study found women were less likely to survive an acute myocardial infarction when treated by a male cardiologist [15]. This disparity in treatment may reflect biases toward women within the cardiology field that could translate to a lack of female participants in cardiology research.

## Conclusions

While the representation of women in medical research has increased over time, it is important that this disparity is not overlooked. The goal of this study was to determine if there has been a difference in rates of female participants within ICD research since the 1980s. Our review shows a sex disparity that has not changed significantly since the introduction of ICDs in the 1980s. The main limitation of this study is that it is a small retrospective study that reviews a limited number of studies. Future studies on the disparity between sexes in ICD trials would benefit from a larger amount of studies and from using more than one journal to pull papers from. A secondary limitation was the “free full text” criteria on PubMed. Future studies would benefit from looking into as many studies as possible, including ones that require fees to access.
